# Supplementary figures and images for: Seasonal effects of long-term warming on ecosystem function and bacterial diversity
Source: PLoS One. 2024 Oct 24;19(10):e0311364. doi: 10.1371/journal.pone.0311364 (PMC11500971; doi:10.1371/journal.pone.0311364)

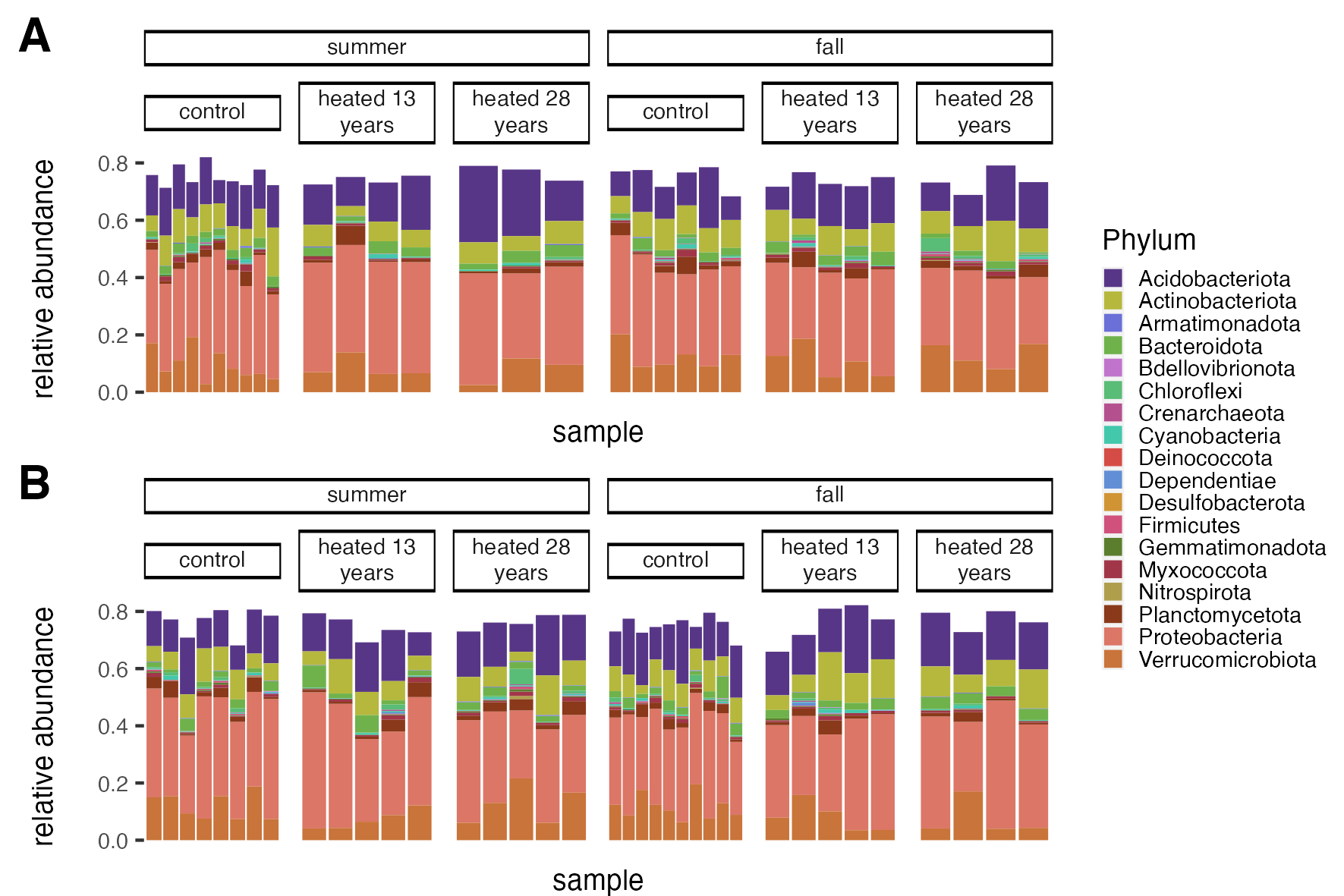

Supplement: S1 Fig — Taxonomy was assigned using SILVA (v 138.1). Sample amplicon sequence variant (ASV) counts were normalized using a sample-specific DESeq2 library size estimation correction factor. Dominant ASVs were determined as ASVs with a relative abundance of 0.001 or higher within a sample. Organic horizon samples are presented in panel A, mineral soil samples are presented in panel B. (tiff) [file pone.0311364.s001.tiff]

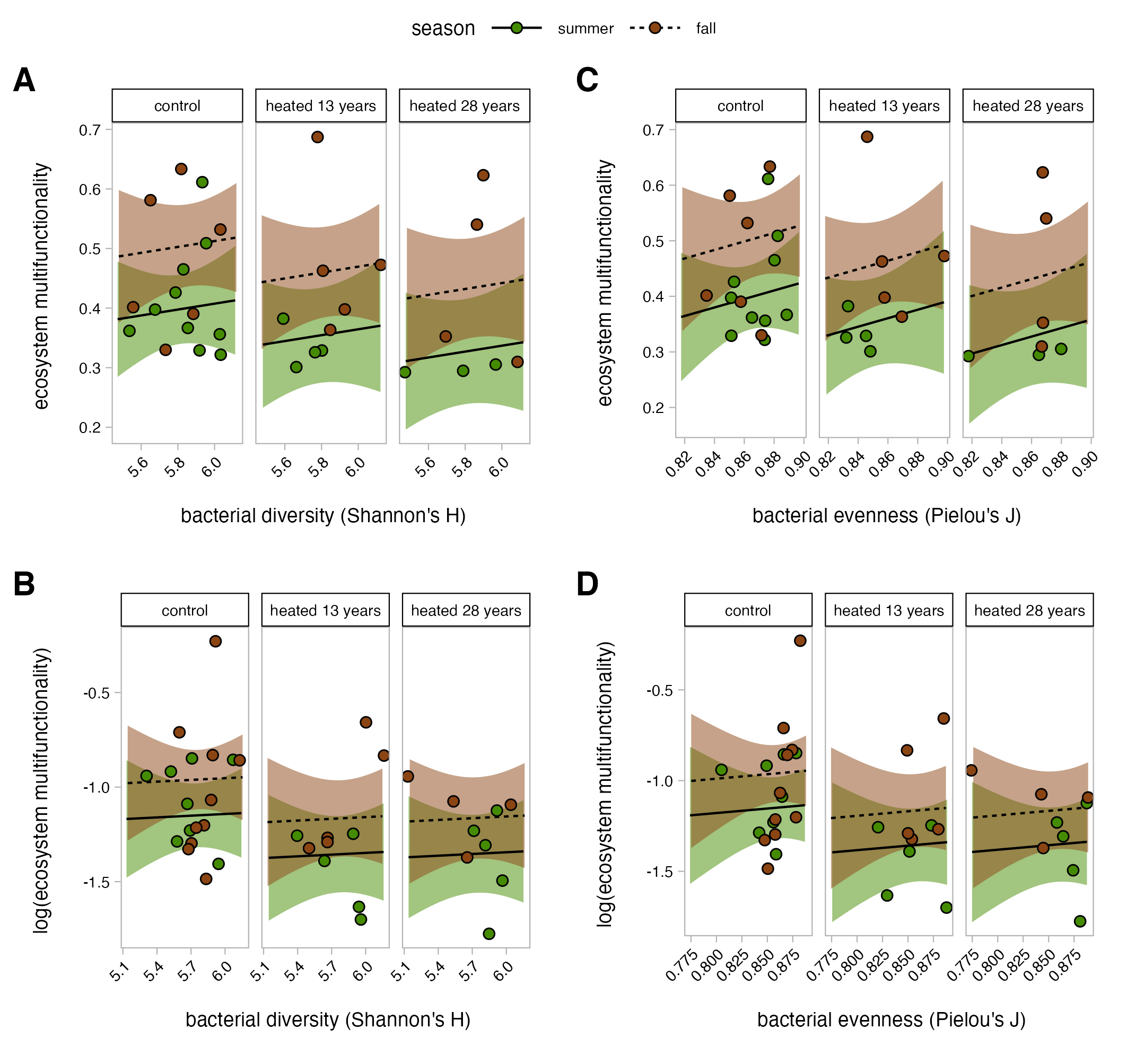

Supplement: S2 Fig — Ecosystem multifunctionality (EMF) was calculated by averaging a set of z-score transformed ecosystem functions or nutrient pools. Mineral soil EMF was log-transformed and is displayed without back-transformation. Bacterial diversity was measured using 16S rRNA gene amplicon sequencing. Shaded regions represent a 95% confidence interval. The reported p-values are Benjamini-Hochberg corrected to account for multiple comparisons. In the organic horizon, fall had a slightly higher intercept compared to summer for both Shannon H (A, p = 0.052) and Pielou J (C, p = 0.055). There was no seasonal trend in the mineral soils (B, D). In the organic horizon and the mineral soils, there was no significant relationship between EMF and bacterial Shannon H or between EMF and bacterial Pielou J, as well as no significant differences between the control, warmed for 13 years, and warmed for 28 years treatments. (tiff) [file pone.0311364.s002.tiff]
