# Supplementary material for: Seasonal effects of long-term warming on ecosystem function and bacterial diversity
Source: PLoS One. 2024 Oct 24;19(10):e0311364. doi: 10.1371/journal.pone.0311364 (PMC11500971; doi:10.1371/journal.pone.0311364)
Supplement: S1 Table — Ecosystem multifunctionality (EMF) and all three bacterial diversity metrics (Shannon H, Chao1 estimated richness, Pielou J) were tested for normality. Variables that were normally distributed (organic horizon Shannon H, Chao1 estimated richness, and Pielou J and mineral soils Shannon H) used a Pearson correlation test, and variables that were not normally distributed (organic horizon EMF and mineral soils EMF, Chao1 estimated richness, and Pielou J) used a Spearman correlation test. (PDF) [file pone.0311364.s003.pdf]

Table S1. Correlation between soil water content and ecosystem multifunctionality and bacterial diversity metrics.
Ecosystem multifunctionality (EMF) and all three bacterial diversity metrics (Shannon's H, Chao1 estimated richness, Pielou's J) were tested for normality. Variables that were normally distributed (organic horizon Shannon's H, Chao1 estimated richness, and Pielou's J and mineral soils Shannon's H) used a Pearson correlation test, and variables that were not normally distributed (organic horizon EMF and mineral soils EMF, Chao1 estimated richness, and Pielou's J) used a Spearman correlation test.

**soil type**	**variable**	**correlation coefficient**	**p value**
Organic horizon	EMF	0.045	0.806
	Shannon's H	-0.065	0.7221
	Chao1 estimated richness	-0.103	0.574
	Pielou's J	-0.006	0.976
Mineral soils	EMF	0.092	0.589
	Shannon's H	-0.084	0.622
	Chao1 estimated richness	-0.180	0.285
	Pielou's J	0.023	0.893
