# Supplementary material for: Seasonal effects of long-term warming on ecosystem function and bacterial diversity
Source: PLoS One. 2024 Oct 24;19(10):e0311364. doi: 10.1371/journal.pone.0311364 (PMC11500971; doi:10.1371/journal.pone.0311364)
Supplement: S2 Table — All models using a Gaussian distribution and model residuals were checked for normality. To meet normality of model residuals, β-glucosidase (BG) activity, respiration, and total nitrogen in the Chao1 model were log-transformed. The model estimates and errors for the ecosystem functions that were log-transformed are reported without back-transformation. All model estimates are reported where the reference for warming treatment is control non-heated plots and the reference for season is summer. Number of samples are reported in parentheses. Errors are reported for a 95% confidence interval. Benjamini-Hochberg adjusted p-values are reported. Any adjusted p-values greater than 0.05 are reported as not significant (n.s.). (PDF) [file pone.0311364.s004.pdf]

Table S1. Single ecosystem function-diversity relationships in the organic horizon. All models using a Gaussian distribution and model residuals were checked for normality. To meet normality of model residuals, β-glucosidase (BG) activity, respiration, and total nitrogen in the Chao1 model were log-transformed. The model estimates and errors for the ecosystem functions that were log-transformed are reported without back-transformation. All model estimates are reported where the reference for warming treatment is control non-heated plots and the reference for season is summer. Number of samples are reported in parentheses. Errors are reported for a 95% confidence interval. Benjamini-Hochberg adjusted p-values are reported. Any adjusted p-values greater than 0.05 are reported as not significant (n.s.).

ecosystem function	diversity metric	predictor	estimate	errors	p value
NAG activity	Shannon diversity	season (15)	-155.666	1958.802	n.s.
		13 years heating (9)	-1846.554	2267.306	n.s.
		28 years heating (7)	-1238.011	2461.991	n.s.
		Shannon (32)	4.701	6029.377	n.s.
	Chao1 estimated richness	season	-121.155	1923.250	n.s.
		13 years heating (9)	-1690.075	2295.138	n.s.
		28 years heating (7)	-1194.214	2445.721	n.s.
		Chao1 (32)	-2.671	8.107	n.s.
BG activity	Shannon diversity	season	-0.520	0.433	n.s.
		13 years heating (9)	0.200	0.502	n.s.
		28 years heating (7)	-0.269	0.545	n.s.
		Shannon (32)	-0.169	1.334	n.s.
	Chao1 estimated richness	season	-0.514	0.417	n.s.
		13 years heating (9)	0.270	0.531	n.s.
		28 years heating (7)	-0.248	229.908	n.s.
		Chao1 (32)	-0.001	0.002	n.s.

Table S1 continued:

ecosystem function	diversity metric	predictor	estimate	errors	p value
Respiration	Shannon diversity	season	0.164	0.276	n.s.
		13 years heating (9)	-0.317	0.320	n.s.
		28 years heating (7)	-0.323	0.347	n.s.
		Shannon (32)	-0.065	0.851	n.s.
	Chao1 estimated richness	season	0.162	0.273	n.s.
		13 years heating (9)	-0.310	0.326	n.s.
		28 years heating (7)	-0.320	0.347	n.s.
		Chao1 (32)	-8.578e-05	0.001	n.s.
Total carbon	Shannon diversity	season	-0.020	0.041	n.s.
		13 years heating (9)	0.063	0.047	n.s.
		28 years heating (7)	0.051	0.051	n.s.
		Shannon (32)	0.140	0.126	n.s.
	Chao1 estimated richness	season	-0.001	0.043	n.s.
		13 years heating (9)	0.005	0.051	0.073
		28 years heating (7)	0.005	0.054	n.s.
		Chao1 (32)	1.118e-04	1.800e-04	n.s.
Total nitrogen	Shannon diversity	season	0.006	0.002	n.s.
		13 years heating (9)	-0.002	0.002	n.s.
		28 years heating (7)	-0.002	0.002	n.s.
		Shannon (32)	0.001	0.006	n.s.
	Chao1 estimated richness	season	0.088	0.188	n.s.
		13 years heating (9)	-0.252	0.224	n.s.
		28 years heating (7)	-0.277	0.122	n.s.
		Chao1 (32)	5.970e-05	0.001	n.s.
