# Supplementary material for: Seasonal effects of long-term warming on ecosystem function and bacterial diversity
Source: PLoS One. 2024 Oct 24;19(10):e0311364. doi: 10.1371/journal.pone.0311364 (PMC11500971; doi:10.1371/journal.pone.0311364)
Supplement: S3 Table — All models used a Gaussian distribution, except total carbon and total nitrogen models, which used an inverse Gaussian distribution with an inverse link function and an inverse Gaussian distribution with an 1/μ2 link function, respectively. Model residuals were examined, and if normality of residuals for the Gaussian models was not met, a log-transformation was applied. Subsequently, N-acetyl glucosaminidase (NAG) was log-transformed to satisfy normality of model residuals. The model estimates and errors for the ecosystem functions that were log-transformed or that used an inverse Gaussian distribution are reported without back-transformation. All model estimates are reported where the reference for warming treatment is control non-heated plots and the reference for season is summer. Number of samples are reported in parentheses. Errors are reported for a 95% confidence interval. Benjamini-Hochberg adjusted p-values are reported. Any adjusted p-values greater than 0.05 are reported as not significant (n.s.). (PDF) [file pone.0311364.s005.pdf]

Table S2. Single ecosystem function-diversity relationships in the organic horizon. All models used a Gaussian distribution, except total carbon and total nitrogen models, which used an inverse Gaussian distribution with an inverse link function and an inverse Gaussian distribution with an $1/\mu^2$ link function, respectively. Model residuals were examined, and if normality of residuals for the Gaussian models was not met, a log-transformation was applied. Subsequently, N-acetyl glucosaminidase (NAG) was log-transformed to satisfy normality of model residuals. The model estimates and errors for the ecosystem functions that were log-transformed or that used an inverse Gaussian distribution are reported without back-transformation. All model estimates are reported where the reference for warming treatment is control non-heated plots and the reference for season is summer. Number of samples are reported in parentheses. Errors are reported for a 95% confidence interval. Benjamini-Hochberg adjusted p-values are reported. Any adjusted p-values greater than 0.05 are reported as not significant (n.s.).

ecosystem function	diversity metric	predictor	estimate	errors	p value
NAG activity	Shannon diversity	season (15)	-0.578	0.555	n.s.
		13 years heating (9)	0.004	0.664	n.s.
		28 years heating (7)	0.163	0.689	n.s.
		Shannon (32)	1.078	1.244	n.s.
	Chao1 estimated richness	season	-0.577	0.565	n.s.
		13 years heating (9)	-0.029	0.680	n.s.
		28 years heating (7)	0.157	0.358	n.s.
		Chao1 (32)	0.001	0.001	n.s.
Respiration	Shannon diversity	season	0.086	0.072	n.s.
		13 years heating (9)	-0.069	0.087	n.s.
		28 years heating (7)	-0.098	0.046	n.s.
		Shannon (32)	0.023	0.083	n.s.
	Chao1 estimated richness	season	0.08635	0.073	n.s.
		13 years heating (9)	-0.070	0.087	n.s.
		28 years heating (7)	-0.098	0.090	n.s.
		Chao1 (32)	2.143e-05	2.115e-04	n.s.

Table S2 continued:

ecosystem function	diversity metric	predictor	estimate	errors	p value
Total carbon	Shannon diversity	season	-5.954	5.749	n.s.
		13 years heating (9)	5.701	6.933	n.s.
		28 years heating (7)	5.451	7.348	n.s.
		Shannon (32)	-4.091	12.893	n.s.
	Chao1 estimated richness	season	-172.024	175.828	n.s.
		13 years heating (9)	156.102	215.646	n.s.
		28 years heating (7)	160.703	232.259	n.s.
		Chao1 (32)	0.085	0.445	n.s.
Total nitrogen	Shannon diversity	season	-44235	55174	n.s.
		13 years heating (9)	48151	67798.36	n.s.
		28 years heating (7)	53284	74613.28	n.s.
		Shannon (32)	-6720	122076.64	n.s.
	Chao1 estimated richness	season	-45373.09	54775.179	n.s.
		13 years heating (9)	46648.91	67727.741	n.s.
		28 years heating (7)	54701.17	74130.512	n.s.
		Chao1 (32)	48.42	165.659	n.s.
