# Supplementary material for: Seasonal effects of long-term warming on ecosystem function and bacterial diversity
Source: PLoS One. 2024 Oct 24;19(10):e0311364. doi: 10.1371/journal.pone.0311364 (PMC11500971; doi:10.1371/journal.pone.0311364)
Supplement: Attachment — Submitted filename: Response to Reviewers.pdf [file pone.0311364.s008.pdf]

We thank both reviewers for their time and helpful comments.

Please note that any references to line numbers refer to the line numbers in the revised manuscript with track changes.

Reviewers' comments:

Reviewer #1: I very much enjoyed reading the paper. It is very well-written and developed, and I particularly appreciate the thorough statistical approach and description. However, I see two major issues.

Firstly, I don't see the value of using a multifunctionality index, as it obscures the individual factors and makes any interpretation in terms of possible mechanisms impossible. Why not use a multivariate approach? Please justify the use of the multifunctionality index in the paper, or consider using a different analytical approach.

Secondly, to enhance the impact of the study, the paper would benefit from the authors explaining why they decided to focus on bacterial diversity only. I miss the context (soil parameters such as nutrient values, soil pH, depth of the organic layer, etc. and a description of the studied forest ecosystem) and a justification for why the impact of the soil fungal communities was disregarded when exploring impacts of the microbial community on the measured ecosystem functions.

Thank you to this reviewer for their comments.

Multifunctionality indices are a widely adopted and validated approach used as an integrative, quantitative measure of the health or robustness of an ecosystem (see lines 44-65). We chose to use this approach to generate a quantitative measure of soil function which can be compared with future work at our site. Additionally, similar to a multivariate approach, the EMF index also aggregates multiple variables (i.e. functions) into a single value (the EMF index). This generates a single set of values, one per sample, that summarizes the trend of all the variables, analogous to a principal coordinate axis for instance. We chose to measure the EMF index for our study to examine the effects of long-term soil warming on the relationship between biodiversity and ecosystem function, a relationship which has been explored and observed in many other ecosystems. While we do agree that with an ecosystem multifunctionality index you cannot distinguish the relationships between individual ecosystem functions and diversity, we did separately investigate the relationships between individual ecosystem functions and diversity (lines 425-437). We found that out of the 7 ecosystem functions included in our EMF index, 6 of the ecosystem functions did not have a significant relationship with any bacterial diversity metric (Table 2, Table 3, Table S2, and Table S3), which is in line with the non-significant relationship between EMF and bacterial diversity metrics. Only β-glucosidase activity had a significant relationship with any of the bacterial diversity metrics (Shannon's H), and this relationship was only significant in the mineral soils. We appreciate the reviewers' comments about the need to investigate or identify the mechanisms (i.e. the selection effect, complementarity) underpinning the biodiversity-ecosystem function relationship. However, this study was not designed to isolate these specific mechanisms. Lastly, we agree that the identification of mechanisms behind the biodiversity-ecosystem function relationship is necessary and valuable for understanding this relationship, but that ultimately was not the primary goal of this study.

We have added additional context about our study site to the methods (lines 112-118).

We wholly agree with the reviewer that fungi are a critical component of the soil microbial community. We did attempt to examine the role of the fungal community on ecosystem function, via ITS2 amplicon sequencing. However, we were only able to successfully PCR amplify 23 out of 80 of our samples. This small subset (<30%) of samples that we did successfully amplify only contained samples from the organic horizon and lacked replicates for each combination season, warming treatment, and warming duration. Thus, we elected to focus only on the bacterial community. We have added this note to the methods (lines 236-240) and some text to this point in the discussion (lines 538-560).

Abstract

"We found that ecosystem function was significantly affected by season, with autumn samples having higher function than summer samples."

Comment: "higher function" - please specify. Do you mean higher process rates?

Here, when we used "higher function" we meant a higher intercept for the EMF diversity model. We have clarified this in the abstract. This sentence now reads, "We found that ecosystem function was significantly affected by season, with autumn samples having a higher intercept than summer samples in our model, suggesting a higher overall baseline of ecosystem function in the fall."

"The effect of warming on bacterial diversity was similarly affected by season, where warming in the summer was associated with decreased bacterial evenness in the organic horizon. Despite the decreased bacterial diversity in the warmed plots, we found that the relationship between ecosystem function and bacterial diversity was unaffected by warming or warming duration."

Comment: These two sentences contain a lot of complex information that is hard to disentangle as is. Instead of using bacterial evenness and bacterial diversity as synonyms (which they aren't entirely), I would suggest sticking with one or the other. Please specify throughout the paper what you refer to when using the term 'diversity' (total number/evenness). Also, in the abstract as well as throughout the paper, the fact that the measured ecosystem functions are not determined solely by bacterial diversity needs to be developed. What are the actual soil parameters such as nutrient values, soil pH, etc.? For instance, in nutrient-poor soils, fungi usually play a dominant role in shaping ecosystem functions and comprise a larger proportion of the total microbial biomass compared to bacterial biomass. What are the biomass proportions of these groups in this study? In other words, the lack of a relationship between ecosystem function and bacterial diversity could be explained by bacteria not playing any significant role in the measured ecosystem functions in these soils, and/or diversity (in terms of species numbers) itself not playing a role if only a few taxa actively contribute to the measured ecosystem functions (expressed as a decrease in evenness).

We have changed bacterial diversity in the second sentence to bacterial evenness. Throughout the manuscript, when we refer to results of bacterial diversity, we have now specified what kind of diversity we are referring to. Since we explored different measures of diversity (Shannon's H, which integrates richness and evenness), richness (Chao1), and evenness (Pielou's J), we were careful to use the word "diversity" when we meant any measure of diversity (H, Chao1, J) or specify which measure when appropriate.

As for the second part of this comment, we agree with the reviewer that ecosystem function is not solely determined by the bacterial community. As mentioned above, we did attempt to sequence

the fungal community. We agree that the ecosystem functions we examined might be driven by fungi and have added a discussion paragraph that addresses this (lines 538-560).

Introduction

Line 27 change "was" to were

Done

Methods

- please add a more detailed description of soil parameters such as, organic layer depth, nutrient content, soil pH, etc. for both sites.

We have added more detail about both sites, including the pH and organic layer depth, to the methods (lines 112-118).

- please add duration between sampling occasion and lab analyses and how samples were treated after sieving in the field

We have added these details to the methods (lines 122-123, 140, 151-152, 162, 168-169, 205-208).

Line 124 please specify "ecosystem functions or soil properties" (x soil properties, x process rates?) I struggle with the term ecosystem functionality because soils are not independent ecosystem but a part of the forest ecosystem

Thank you for this comment. We have revised this sentence to better reflect the idea that soils are a component of the forest ecosystem rather than an ecosystem themselves (line 136).

Results

Line 333 What does this mean "In the organic horizon, EMF was significantly higher" given that multifunctionality is a function of both process rates and nutrient pools? If any of the values contributing to EMF change, EMF will change in some way. How can EMF be interpreted without knowing the input values. Can you please add the information of the input values not just their statistical response as presented in Table S1 & Table S2

We have added the estimates and errors to the written results when values are not available in a table (lines 396, 411, 413-414). Additionally, the data are publicly available in the Harvard Forest Data Archive.

Line 343 what is EMF-diversity?

We were referring to the relationship between EMF and bacterial Chao1 estimated richness. We have revised this sentence to clarify this (line 409).

Discussion

The discussion is comprehensive and well written. The role of the fungal community needs to be addressed throughout the paper.

Thank you. We have added a paragraph in the discussion about fungal contributions to the soil functions we studied (lines 538-560).

Line 482 add citation

Thank you for pointing this out, we have added a citation (line 573).

Reviewer #2: The main aim of the manuscript is to analyze effects of season, soil depth and long-term soil warming on soil bacterial community in temperate deciduous forest using the analysis of relationship between bacterial alpha diversity (assessed by 16S rRNA gene amplicon sequencing) and ecosystem multifunctionality (using environmental parameters such as enzyme activity, microbial biomass, soil respiration and soil C and N). The manuscript deals with interesting topic and uses the soil warming experimental site which is valuable due to the long-term running and that has been widely studied before. However I have several concerns regarding the manuscript. First of all the authors did not measure soil moisture and soil pH at the studied site, which are very likely the most important environmental parameters shaping microbial communities. Furthermore often results (either data or statistical evidence) that are claimed in the manuscript are not shown-neither in the text nor figures nor tables. Also certain information is referred to be in specific figure or table, but it is not possible to see it there. Also I miss the access to the original data (sequence data, enzyme activity or soil properties). See below more specific comments:

Thank you to this reviewer for their comments.

While we did not report pH for these samples, previous work at the experimental site has documented no significant differences in pH between the different experimental treatments or soil layer (see Anthony et al. (2020), Pec et al. (2021), and Pold et al. (2017)). We have added this detail to the methods along with previously reported pH values (lines 114-116).

We agree with the reviewer that moisture can drive microbial activity, and long-term warming does affect moisture (see Contosta et al. (2015) and Domeignoz-Horta et al. (2023)) and water holding capacity (see Werner et al. (2020). We did measure moisture in this sampling campaign; however, the ecosystem functions measured in the lab (e.g. enzyme activity, respiration) were performed under the same water content to exclude the indirect effect of moisture on the measurements. In response to this reviewer's comment, we conducted correlation tests to examine whether percent soil water content was correlated with any of the three bacterial diversity metrics as well as EMF. We found that there was no significant correlation between percent soil water content and EMF, bacterial Shannon diversity, Chao1 estimated richness, or evenness (Pielou's J) in either the organic horizon or mineral soils and have reported the results in a new supplementary table (Table S1).

As for access to the original data, the bacterial sequencing data was under embargo, but is available now on the NCBI SRA (Accession ID: PRJNA957455). Five out of seven of the ecosystem functions (respiration, microbial biomass, β-glucosidase activity, oxidative enzyme activity, and N-acetyl glucosaminidase activity) in this study were measured as part of a previous study and those data are publicly available via the Harvard Forest Data Archive (Dataset ID: HF431, doi:10.6073/pasta/ade36a6a8388a32a0dd6e7754b4e997b). The total soil organic carbon data, total nitrogen data, z-score transformed ecosystem function measurements, and ecosystem multifunctionality index values are available in the supplement.

Specific comments:

Line 6 . . . diversity of what?

All three references in this sentence looked at microbial diversity. The sentence has been revised to clarify that (line 6).

Line 11-14. . . . it is true, but there are many studies available that used in-situ climate manipulations to analyze effect of warming on the soil ecosystem (including microbial communities)

We apologize for not being clear. Yes, there are many studies that examine the effects of warming on the soil ecosystem, including microbial communities, but we are referring specifically to studies that examine how warming affects the relationship between biodiversity and ecosystem function. For this specific question, there are fewer studies, especially when considering whether these studies used non-laboratory systems or non-manipulated diversity. We have rewritten this section to clarify this (lines 12-16).

Line 66 . . . what is meant by active layer of soil? Active layer is the term used in permafrost affected soil

We apologize for the confusion. We were referring to the metabolically active layer of the soil in this case. However, to avoid any confusion, we have revised this sentence (line 68). This sentence now reads: "Long-term warming increases soil CO_2 efflux, and results in faster upticks in soil respiration after the spring thaw."

Line 108. . . what was the diameter of soil cores?

The cores were taken with a 5.7 cm diameter tulip bulb corer. We have added this information to the methods (line 119).

Line 124. . . . I count 8 different functions, not 7

The oxidative enzyme activity assay we used was based on the substrate L-DOPA plus H2O2, and measures both phenol oxidase and peroxidase activity in the same assay. This activity is reported as oxidative enzyme activity. This detail has been added to the manuscript to clarify (lines 185-187).

Line 167-168. . . you write that organic and mineral horizons were analyzed separately, what about samples collected in different seasons?

We were interested in examining seasonal influences on the EMF-diversity relationship, so we could not analyze the summer and fall samples separately. We separated the organic and mineral soils since we already know that these two soil layers are distinct from one another (see DeAngelis et al. (2015) and Pold et al. (2017))

Line 222-225. . . explain what issues with sequencing you had. . . . It is not clear from the description what is meant by the replicates here ? Is it related to original 5 replicates site for each sample?

We have revised this sentence to clarify what replicates we are referring to (lines 261, 263).

For some of the samples, we were unable to successfully PCR amplify them during the library preparation step. We have added this detail to the methods (lines 258-260).

Line 243-244... what parameters were tested by Wilcoxon and t-test? For t-test were the data normally distributed?

All parameters were checked for normality using a Shapiro-Wilk test. EMF values for both the organic horizon and mineral soils were not normally distributed, and so differences between the control plots' EMF were tested for using a Wilcoxon rank sum test. The bacterial diversity metrics for both the organic horizon and mineral soils were normally distributed, and so differences between the control plots' bacterial diversity were tested with a t-test. We have added more detail to the methods to clarify this (lines 282-289).

Line 252... soil type is meant soil horizon?

Yes, we used soil type instead of soil horizon since our sampling design sampled to a depth of 10 cm from the soil surface, which at our site captures all of the organic horizon, but not all of the A horizon in the mineral soil. We have changed this to soil layer to avoid confusion (line 304).

Line 303-304... why only organic horizon and summer samples were tested ?

We examined only the organic horizon summer samples since those were the only samples that had a significant difference in any bacterial diversity metric between the heated and the control treatment. We have added some text to make this clear (lines 350-352).

Line 308....relative abundance threshold is related to entire sequence dataset or individual samples from summer and organic horizon?

The relative abundance threshold was based on individual samples. We have added that to clarify (lines 365).

Line 309-310... is this related to entire dataset or to summer and organic horizon samples only?

This is related to the organic horizon summer samples only. We have added that for clarification (line 366).

Line 316 ... I am unable to see the stated result in Table 1.

The results that support this sentence are the significant interaction between warming and season for Pielou's J in Table 1. As referenced in the methods, any significant interactions were investigated using a Tukey's HSD test to identify which combination of factors (i.e. warming treatment, warming duration, and/or season) were significantly different (lines 350-352). We have also added the results from the Tukey HSD test on the interaction between season and warming treatment for Pielou's J in the results (lines 374-375).

Line 327... chao1 increase by 12% is not evident from Table 1.

We apologize for this typo. We have revised the stated change to address this typo (lines 386-387).

Line 332... can you report any specific results of statistical analysis that would prove such statement?

We have revised this sentence to clarify that season had a significant effect on EMF and that

warming treatment or warming duration had no effect on EMF (lines 394-395).

Line 335... what means somewhat higher? Be specific, all results you claim has to be supported by statistical analysis which either shows that something was significantly higher or the difference was non-significant

In the methods we outlined that due to the uniqueness of the samples in this study, we elected to report results with adjusted p-values ≤ 0.05 as significant and results with adjusted p-values between 0.05 and 0.1 as trends (line 354-355). We have clarified that this is not a statistically significant trend (line 398).

Line 343-344.... in the figure I cannot see any evidence of significant difference that is stated in the text....

To clarify, this result is irrespective of warming treatment/duration (lines 410-411). We have also added the estimates and a 95 % confidence interval to the text (lines 411, 413-414).

Line 369-371... where are these data shown? Where is the evidence for significant changes that are stated?

We have added a reference to the table that contains the data that support these sentences (line 437).

Fig1 description A) Was the EMF from mineral horizon really log transformed as is stated in figure description, if so add this info to the y axis description.

Thank you for pointing this out. This is a typo in the figure legend. We did log-transform the mineral soils' EMF when we constructed our general linear models for the statistical analyses, but we have plotted the original values for the figure. We have revised the figure legend to clarify this.

B) the description says that there are p-values reported in the figure- I cannot see them, please explain where is possible to see p-values in the figure.

Our apologies, we were referring to the p-values reported in the figure legend. We have removed the sentence that stated the p-values as well as the sentence describing what p-values we report.

Fig 2 description In the figure description explain what is on the figure, do not write the results, they should be obvious from the figure or written in the result part of the manuscript.

Thank you for this comment. We have removed the sentences that refer to results from both the legends for figure 1 and figure 2.
