# Supplementary material for: Seasonal effects of long-term warming on ecosystem function and bacterial diversity
Source: PLoS One. 2024 Oct 24;19(10):e0311364. doi: 10.1371/journal.pone.0311364 (PMC11500971; doi:10.1371/journal.pone.0311364)
Supplement: Attachment — Submitted filename: Response to Reviewers.pdf [file pone.0311364.s009.pdf]

Thank you again to the reviewers and editors for your assistance and comments.

Specifically: Throughout list of references, use journal name abbreviations; please check all references and adjust accordingly References 5, 13, 27 and 41: Please insert volume/pages/article number

We have abbreviated all of the journal names following NCBI abbreviations.

Reference 5 now has the updated year, volume number, article number, and page numbers.

Reference 13 has been updated to include the page numbers.

Reference 27 now has the volume and article number as well as the page numbers.

Reference 41 now has the page numbers.

Reviewer 1: I very much enjoyed working with the authors of this manuscript. It was a constructive and productive process. The responses to all of the reviewers' comments were thorough and thoughtful. I appreciate the discussion about the possible role of the soil fungal community, as well as the honest explanation of why it was not included in the study. I have no further comments and recommend this study for publication in PLOS ONE. Many thanks from me!

Thank you for your comments!

Reviewer 2: The authors addressed the raised concerns and answered the questions asked. I would recommend to double check the references, especially capitalization of names of journals should be unified.

Thank you for pointing this out. We have corrected the capitalization in the journal names in the references.
